# Nanoparticle-Based Radiosensitization

**DOI:** 10.3390/ijms23094936

**Published:** 2022-04-29

**Authors:** Ivan Kempson

**Affiliations:** Future Industries Institute, University of South Australia, Mawson Lakes 5095, Australia; ivan.kempson@unisa.edu.au

Radiotherapy is a highly affordable treatment and provides many excellent outcomes. However, a degree of desperation persists in driving innovation and improvements in the delivery of radiotherapy to provide better treatment outcomes for difficult-to-treat cancers. In this regard, sensitizers of tumor have been pursued and metal nanoparticles have advanced beyond the laboratory to being clinically available. Nanoparticles offer a range of advantages, not only from a scientific perspective, but also from a health economics perspective, as nanoparticles are relatively cheap compositionally and in their manufacture. The additional cost burden of metal nanoparticles can be relatively easily absorbed compared to other emerging treatments with exorbitant costs that will not feasibly be supported by public health systems. In this regard, nanoparticles present themselves as being relatively easily adopted into current radiotherapy practices and to be accessible for the general public where radiotherapy infrastructure exists.

This Special Issue, titled Nanoparticle-Based Radiosensitization 2.0, was delivered on the back of the success of the first instalment [1] in this series dedicated to potentiating radiotherapies with nanoparticles. The content of this Special Issue presents this concept in the current environment where basic, applied, and clinical research continues with a momentum that is increasingly identifying and quantifying fundamental mechanisms [2], improving understanding of structure–function relationships to aid quality-by-design [3] and potential clinical outcomes [4]. 

Once again, this Special Issue highlights the multidisciplinary and complex field of research. Even in the fundamental research of nanoparticle radiosensitization, a generic understanding of the mechanisms remains elusive. This is highlighted by the observations reported by Marie Hullo and Emmanuelle Bourneuf et al. [5] who identify that vastly different radiobiological responses result between cell lines under identical experimental conditions. The inclusion of computational modelling in their work highlighted the inability to explain the observations based on localized dose enhancement. Physical mechanisms did not account for the intercellular variability and the chemical and biological mechanisms, whether due to variability in nanoparticle uptake or propensity, which led to certain cell lines having a vulnerability to sensitization, while others did not. However, it is only recently stipulated that computational models have begun to include the generation of reactive oxygen species in nanoparticle radiosensitization studies [6]. Radiolysis yield enhancement adds to mechanistic description of radiosensitization. This is exemplified in the work led by Prof Eva Bezak where Dylan Peukert et al. modelled the generation and diffusion of reactive oxygen species, resulting in proton irradiation of gold nanoparticle inside a virtual cell [7]. This modelling contributes to the evidence that nanoparticles can enhance proton therapeutic effects via a generation of reactive species. The work further shows the spatial range of these species is very limited and that nanoparticles ideally need to be proximally located adjacent to sub-cellular targets to have a major impact. DNA damage enhancement due to ROS was only theoretically significant if the nanoparticles were accumulated at the nuclear membrane (assuming that they will generally not penetrate the nucleus). The range of reactive species also indicates that radio enhancement through increasing ROS is probably only limited to cells with internalized nanoparticles, as the range of ROS is too short to reach adjacent cells. 

Further variables critical to the degree of enhancement with respect to physical mechanisms were thoroughly investigated by the team led by Dr. Vladimir Morozov. They modelled dose enhancement factors for a comprehensive set of elemental compositions and kV X-ray spectra [8]. Very interestingly, they also looked at the difference in dose enhancement that can occur with spectral changes as an X-ray beam traverses the tissue. The variability between various nanoparticle compositions and spectral variations, depending on variables such as tube voltage, filtering, and depth in phantoms, can provide a wealth of information for contemplating an optimal nanoparticle and treatment modality. 

Regardless of mechanisms, there is often an assumption that nanoparticles can be delivered efficaciously. In the work overseen by Prof Pai-Chi Li, an innovative test of applying localized sonoporation to enhance nanoparticle uptake was performed [9]. This treatment enhanced penetration of nanoparticles into cells and, when combined with irradiation from a clinical 6MV photon source, the nanoparticles increased the γH2AX foci and reduced clonogenic survival. The results also translated into a pre-clinical model whereby tumor growth was substantially impeded. The work highlighted a concept whereby therapeutic doses can be easily achieved with less formulation, thereby reducing nanoparticle dosage and potential toxicities.

In vivo work continues to identify further complexities in understanding radiosensitization mechanisms. A study led by Prof Nohyun Lee and Prof Hee Chul Park [10] highlights the importance of considering hypoxia, which often presents clinically but is rarely considered in in vitro assessment. Hypoxia is a major antagonistic factor in radiotherapy outcomes which nanoparticles may overcome. Their work also emphasized the importance of other biological mechanisms that will only be observable with pre-clinical studies in immunocompetent animals. Specifically, they reported tumor cell population manipulation and remodeling by their nanoparticle. Regardless of the use of ionizing radiation, the nanoparticle increased the number of cytotoxic T-cells. Radiation enhanced this oncolytic phenomenon and highlights the interesting effects that nanoparticles may have on remodeling the tumor microenvironment by inducing a beneficial immune response.

The overall therapeutic effect from nanoparticle radiosensitization is dependent on a range of mechanisms, including physical, chemical, and biological mechanisms. These reports highlight that it is not just a matter of how radiation interacts with nanoparticles, but also how nanoparticles interact with cells that give rise to therapeutic effects.
